# Correction: Synergistic adsorption of ciprofloxacin on an amine-functionalized Zn-MOF/chitosan–polyethylenimine composite sponge: experimental and statistical optimization and biological activity

**DOI:** 10.1039/d6ra90071b

**Published:** 2026-07-15

**Authors:** Meshari M. Aljohani, Sharifah G. Alharthi, Aliyah S. Alhawiti, Nada Alkhathami, Halimah Alahmari, Asma O. Obaid, Nashwa M. El-Metwaly

**Affiliations:** a Department of Chemistry, College of Science, University of Tabuk 71474 Tabuk Saudi Arabia; b Department of Chemistry, College of Science, University of Bisha P.O. Box 551 Bisha 61922 Saudi Arabia; c Department of Chemistry, College of Science and Humanities in Al-Kharj, Prince Sattam Bin Abdulaziz University Al-Kharj 11942 Saudi Arabia; d Department of Physical Sciences, Chemistry Division, College of Science, Jazan University P.O. Box. 114 Jazan 45142 Saudi Arabia; e Department of Chemistry, College of Science, Umm Al-Qura University Makkah 24230 Saudi Arabia nmmohamed@uqu.edu.sa n_elmetwaly00@yahoo.com

## Abstract

Correction for ‘Synergistic adsorption of ciprofloxacin on an amine-functionalized Zn-MOF/chitosan–polyethylenimine composite sponge: experimental and statistical optimization and biological activity’ by Meshari M. Aljohani *et al.*, *RSC Adv.*, 2026, https://doi.org/10.1039/d6ra03353a.

The authors regret that the one of the affiliations (affiliation *e*) was incorrectly shown in the original manuscript. The corrected list of affiliations is as shown herein.

The Royal Society of Chemistry apologises for these errors and any consequent inconvenience to authors and readers.

